# Anaerobic Co-digestion of Rice Straw and Pig Manure Pretreated With a Cellulolytic Microflora: Methane Yield Evaluation and Kinetics Analysis

**DOI:** 10.3389/fbioe.2020.579405

**Published:** 2021-02-04

**Authors:** Bin Zhong, Xuejiao An, Fei Shen, Weijuan An, Qinghua Zhang

**Affiliations:** Jiangxi Engineering Laboratory for the Development and Utilization of Agricultural Microbial Resources, College of Bioscience and Biotechnology, Jiangxi Agricultural University, Nanchang, China

**Keywords:** agricultural waste, biological pretreatment, anaerobic co-digestion, methane, kinetics, energy balance

## Abstract

Agricultural wastes, such as rice straw (RS) and pig manure (PM), cause serious environmental pollution due to the non-existence of effective disposal methods. Urgent investigations are needed to explore how such wastes can be transformed into resources. In this study, we comprehensively assessed methane yield and kinetics of RS and PM anaerobic co-digestion, with or without pretreatment of a previously developed cellulolytic microflora, under conditions of their maximum organic loading rate. The anaerobic co-digestion results revealed that the cumulative methane production of RS and PM after bio-pretreatment was 342.35 ml (g-VS)^−1^, which is 45% higher than that of the control group [236.03 ml·(g-VS)^−1^]. Moreover, the kinetic analysis showed the first-order kinetic, while the modified Gompertz models revealed higher fitting properties (*R*^2^ ≥ 0.966). After bio-pretreatment, the hydrolytic constant, maximum accumulative methane production, and maximum methane production rates of RS and PM reached 0.46 day^−1^, 350.79 ml·(g-VS)^−1^, and 45.36 ml·(g-VS)^−1^·day^−1^, respectively, which were 77, 45.1, and 84.3% higher than those without pretreatment. Also, we found that the lag phase and effective methane production time after bio-pretreatment decreased from 2.43 to 1.79 days and 10.7 to 8.92 days, respectively. Upon energy balance evaluation, we reported a net energy output of 5133.02 kWh·ton^−1^ after bio-pretreatment. Findings from this present study demonstrated that bio-pretreatment of RS and PM mixtures with cellulolytic microflora could greatly enhance methane production and anaerobic digestion efficiency.

**Graphical Abstract:**
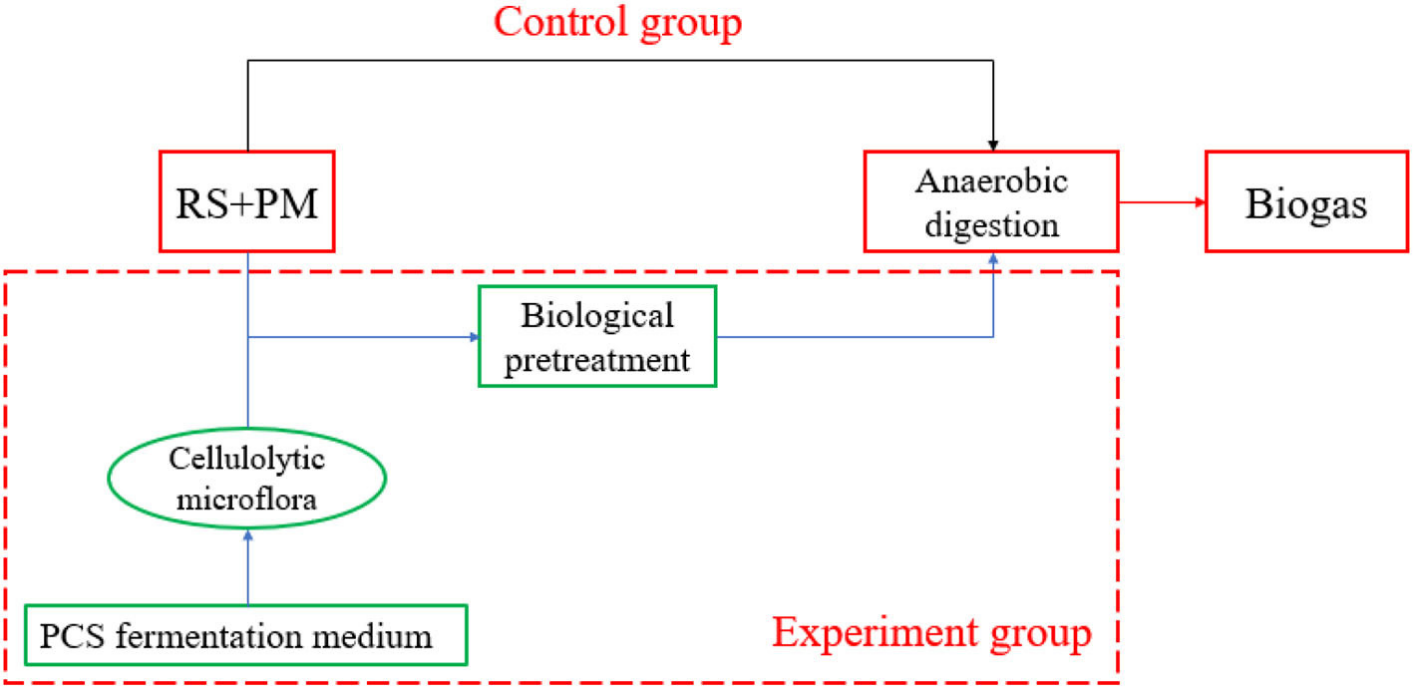
Flowchart with the followed procedure and methods.

## Introduction

As a traditional agricultural country, nearly 29.69 million hectares of land in China is under rice production. In 2019, for instance, the rice output was 209.61 million tons (National Bureau of Statistics, 2019). Notably, due to the high grass-to-valley ratio (1–1.5) of rice (Kainthola et al., 2019), a large amount of rice straw (RS) is produced. On the other hand, with the large-scale development of pig farming in China, the environmental pressure brought by pig manure (PM) is gradually increasing (Wang et al., 2018). RS and PM are thus regarded as two types of typical agricultural wastes causing environmental pollution owing to the lack of efficient disposal measures (Chelme-Ayala et al., 2011; Yin et al., 2014). In previous studies, most of these wastes were processed either into feedstuffs or fertilizers (Qian et al., 2014); however, their utilization efficiencies remained relatively low. Therefore, there is an urgent need to explore new approaches to improve the utilization efficiency of these agricultural wastes.

Based on the current understanding, the bioconversion of these agricultural wastes into methane has attracted increasing attention across the globe (Deepanraj et al., 2015; Zhu et al., 2015; Andrew et al., 2018) for the advantages of low energy consumption, low equipment requirements, and milder operating conditions. It is generally believed that crop straw is rich in lignocellulosic content and a resultant high carbon/nitrogen (C/N) ratio, while this ratio in animal manure is relatively lower for its rich nitrogen content. Therefore, the direct digestion of these types of substrates will result in the lower conversion efficiency of organic carbon or ammonia accumulation (Yangin and Ozturk, 2013). To curb these drawbacks, mixing two or more types of substrates into an anaerobic digestion (AD) system (i.e., co-digestion) is regarded as one of the simple and acceptable strategies, which has received some positive impacts as highlighted by literature reports (Gopi et al., 2014; Logan and Visvanathan, 2019; Villa Gomez et al., 2019). However, the recalcitrant lignocellulosic structures of the substrates will still restrict the hydrolysis efficiency and further result in a lower methane yield (Karimi et al., 2013). Therefore, it is necessary to break up these recalcitrant structures using pretreatment strategies before AD. Compared with physicochemical methods, bio-pretreatment, especially through microbial co-culture methods (i.e., microflora), is more advantageous in terms of high enzymatic activity and lack of metabolite repression and feedback regulation problems (Haruta et al., 2002) and has attracted far much attention in recent AD research (Hu et al., 2016; Ali et al., 2017; Kong et al., 2018).

To further comprehend an AD system and predict its methane production, it is necessary to analyze the kinetics of methane production and evaluate the anaerobic performance. Generally, it is believed that AD has four phases, including hydrolysis, acidogenesis, acetogenesis, and methanogenesis. Currently, hydrolysis and methanogenesis have widely been investigated because most researchers believe that these phases greatly limit the dynamic process of AD (Taricska et al., 2011). In recent decades, numerous modeling studies using the modified Gompertz model, ADM1 model (Anaerobic Digestion Model No. 1), first-order kinetic model, cone model, Weibull model, and others have been carried out to characterize AD processes (Zhang et al., 2015; Lyu et al., 2019; Nguyen et al., 2019; Villamil et al., 2019). Among these models, the first-order kinetic model, regarded the most classical, has been widely used in systems involving complex wastes due to its simplicity and practicability (Mata-Alvarez et al., 1993). It could be used on the hypothesis that the hydrolysis phase limits the AD process, and meanwhile, the lag phase will not be considered. Also, it was reported that with the use of lignocellulose substrates such as stalks, the first-order kinetic model could efficiently describe the AD process (Lo et al., 2010). Furthermore, the cumulative methane production in the AD process is believed to conform to the sigmoidal curve, and such a process owns a significant lag phase stage. The modified Gompertz model, which has widely been considered to be the most suitable in describing the sigmoidal curve, can better fit the relationship between cumulative methane production and time in batch AD experiments (Zhai et al., 2015; Luz et al., 2018). Many researchers have received some positive results by applying these models to estimate methane production from AD using different substrates (Shen and Zhu, 2016; Li et al., 2018; Zahan et al., 2018). Although reports on anaerobic co-digestion with different substrates using different pretreatment strategies have been well-documented (Liu et al., 2014; Mustafa et al., 2016; Zhao et al., 2018), relevant literature reports that comparatively investigated the methane yields and kinetics of RS and PM anaerobic co-digestion with or without bio-pretreatment are scarce. Although previous studies have determined the feasibility of biological pretreatment (Shen et al., 2018), it is inaccurate to directly compare the AD data in different studies due to differences in substrates and processing methods. By establishing a mathematical model to predict the performance of AD with and without biological pretreatment, and comparing the kinetic parameters of AD in other studies, the feasibility of the pretreatment strategy can be better evaluated. Additionally, bio-pretreatment is an environmentally friendly pretreatment method, and a few reports exist on the energy balance and AD. Rodriguez's research shows that biological pretreatment can increase methane yield by 30–50% and can generate 834 kW of net energy (Rodriguez et al., 2017).

In the present study, RS and PM anaerobic co-digestion was conducted with or without bio-pretreatment using a previously constructed cellulolytic microflora. The main genera that synergistically degrade cellulose in the microflora are *Clostridium, Petrobacter, Defluviitalea*, and *Paenibacillus* (Zhang et al., 2011a), among them, *Clostridium* is the key genera that degrade cellulose, which could efficiently disintegrate filter paper via the secretion of cellulose-binding proteins (Zhang et al., 2011a). The aims of this study are (i) to investigate the effect of cellulolytic microflora on methane yield in RS and PM AD system, (ii) to establish the first-order kinetic and modified Gompertz models to accurately predict methane production and assess the relationship between AD process and kinetic parameters, and (iii) to determine the economic feasibility of applying composite microbial pretreatment substrates, whereby the energy production and consumption were balanced. Moreover, we used kinetic parameters to provide technical guidance for large-scale AD of RS and PM and to provide a basis for pretreating other fibrous agricultural wastes.

## Materials and Methods

### Substrates and Anaerobic Sludge

RS and PM were collected from Jiangxi Agricultural University, Nanchang, China. The straw was dried and crushed using a solid crusher but was not sieved. PM was placed in the refrigerator at 4°C, during which no other treatment was done. The major components of RS and PM were as follows (Table 1).

**Table 1:** The major components of raw materials.

	**Rice straw**	**Pig manure**	**Sludge**
Total carbon (%)	36.8 ± 1.21	7.95 ± 1.12	–
Total nitrogen (%)	0.623 ± 0.03	1.03 ± 0.04	–
Total hydrogen (%)	5.45 ± 0.18	5.52 ± 0.21	–
Total oxygen (%)	30 ± 1.42	31 ± 1.68	–
Total solids (TS, %)	–	–	10.20 ± 0.31
Volatile solids (VS, %)	–	–	5.22 ± 0.04

*± the following value represents the standard deviation of the measurement, obtained as average values of three replicates*.

Expressed as weight percent on a dry basis.

*–, represents not determined*.

### Activation of the Cellulolytic Microflora

A peptone–cellulose solution (PCS) medium containing 5.0 g of filter paper (the round medium-speed qualitative filter paper) with a diameter of 12.5 cm was used. For convenience, it was cut into a rectangular shape measuring 1 × 10 cm. Then, 0.9 g of CaCO_3_, 5.0 g of NaCl, 5.0 g of peptone, 1.0 g of yeast extract, 1.8 g of PM, and 1 L of water (pH 7.6) were added to activate the cellulolytic microflora, which was isolated from decaying straw and silt in nature and the filter paper could be completely decomposed at 55°C under 40 h of incubation by secreting cellulose-binding proteins (CBPs) (Zhang et al., 2011a, 2016). A total of 10% (v/v) of the microflora solution was inoculated into the PCS fermentation medium and incubated at 55°C under static conditions for 36 h.

### Biological Pretreatment

A total of 99 g RS (51.15 g) and PM (47.85 g) mixture with a C/N ratio of 30:1 was combined at the biological pretreatment stage. Thereafter, the activated microflora solution was inoculated at the ratio of 10% (v/v) and supplemented with water to achieve a total volume of 1,000 ml, where pH was adjusted to 7.6 using 2 mol·L^−1^ NaOH solution. All these mixtures were incubated at 55°C under static conditions for 30 h. The above bio-pretreatment experiments were performed in triplicate for the subsequent AD.

### AD Design

The whole study was conducted in nine parallel AD reactors with 9-L working volume, and a 2-L free space was left at the top of the reactor for gas generation. A similar amount (300 g) of anaerobic sludge collected by centrifugation (8,000 r·min^−1^, 10 min) was inoculated into each reactor and mixed with 9 L of water. Thereafter, the bio-pretreated RS and PM mixtures with a cellulolytic microflora were transferred into three AD reactors at a maximum organic loading rate [OLR, 2.5 kg COD/(m^3^·day)], in which the maximum accumulative methane production and maximum methane production rate were the highest. Meanwhile, the control groups (without biological pretreatment) were set up by adding RS and PM mixtures with equal volume of sterilized cellulolytic microflora solution into another three reactors using the same OLR condition. Furthermore, the blank groups (CK) were carried out in the remaining three AD reactors, where we only evaluated the methane produced from a mixture of 9 L of water and 300 g of anaerobic sludge. Each reactor was purged with N_2_ gas for 5 min before sealing with rubber gaskets to maintain the anaerobic condition and mesophilic (35 ± 0.5°C) operating condition for 15–20 days. Moreover, the stirring speed of these reactors was maintained at 30 r·min^−1^. To observe the changes in AD parameters, a small amount of supernatant from fermentation broth was collected via centrifugation (8,000 r·min^−1^, 10 min) every day. Detailed information on the mounts of the substrates added with or without biological pretreatment is summarized in Table 2.

**Table 2:** Details of the additional amounts of substrates.

**OLR (g COD·L^**-1**^**·**day^**-1**^)**	**Control group**			**Experimental group**			
	**RS (g)**	**PM (g)**	**Water (ml)**	**RS (g)**	**PM (g)**	**Water (ml)**	**Cellulolytic microflora solution (ml)**
2.5	34.93	32.67	1,000	51.15	47.85	900	100

### Calculating the Accumulative Methane Production and Methane Production Rate

Methane produced from RS and PM mixtures with or without biological pretreatment was determined through the water displacement method, where the carbon dioxide and H_2_S were removed using 2 mol·L^−1^ of NaOH solution and the volume of the discharged NaOH solution was equivalent to the methane (Zhang et al., 2011b). Furthermore, the volume of methane produced by the sludge inoculums (i.e., blank group) was deducted from the entire volume produced by RS and PM with or without biological pretreatment. The rate of methane production and accumulated methane production were calculated as follows:

(1) $$\begin{array}{l} R\left(t\right)\;=\;\frac{V_{t}\;-{\;V}_{\text{CK},\text{ t}}}{\text{VS of RS and PM mixtures added}} \end{array}$$

(2) $$\begin{array}{l} M\left(t\right)\;=\overset{t}{\underset{t=1}{\sum }}\frac{V_{t}\;-{\;V}_{\text{CK},\text{ t}}}{\text{VS of RS and PM mixtures added}} \end{array}$$

where *R*(*t*) denotes the methane production rate [(ml·(g-VS)^−1^·day^−1^] at AD time *t* (days), *V*_*t*_ denotes the methane volume (ml) of RS and PM with or without biological pretreatment at AD time *t* (days), *V*_CK,t_ represents the methane volume (ml) of sludge inoculum at AD time *t* (days), and *M*(*t*) denotes the cumulative methane production [ml·(g-VS)^−1^] at AD time *t* (days).

### Calculating the Theoretical Methane Yield and Biodegradability

The theoretical biochemical methane potential (TBMP) under standard conditions (0°C, 1 bar) was assessed based on the elemental composition of the substrates, according to Buswell's formula:

(3) $$\begin{array}{l} {\text{C}}_{\text{c}}{\text{H}}_{\text{h}}{\text{O}}_{\text{o}}{\text{N}}_{\text{n}}+\left(\frac{4c-h-2o+3n}{4}\right){\text{H}}_{2}\text{O}\\ =\left(\frac{4c+h-2o-3n}{8}\right)\text{C}{\text{H}}_{4}+\left(\frac{4c-h+2o+3n}{8}\right)\text{C}{\text{O}}_{2}+\text{nN}{\text{H}}_{3} \end{array}$$

(4) $$\begin{array}{l} CH_{4}^{TBMP}\left(\text{ml C}{\text{H}}_{4}/\text{g VS}\right)\\ =22.4\times \left[\left(\frac{4c+h-2o-3n}{8}\right)/\left(12c+h+16o+14n\right)\right]\times 1000 \end{array}$$

The substrate biodegradability was calculated according to Equation (5) (James et al., 2014):

(5) $$\begin{array}{l} \text{Biodegradability}\\ \text{=}\frac{\text{cumulative methane production }(\text{ml/g VS})}{\text{theoretical methane production }(\text{ml/g VS})}\;\times \text{100}\% \end{array}$$

### Kinetic Assessment

The first-order kinetic model (Equation 6) was used to describe the hydrolysis constant (Liu et al., 2015).

(6) $$M(t)=M_{\text{max}}\cdot [1-\text{exp}(-kt)]$$

where *M*(*t*) denotes the cumulative methane production [ml·(g-VS)^−1^] at AD time *t* (days), *M*_max_ denotes the maximum cumulative methane production potential [ml·(g-VS)^−1^], and *k* denotes the hydrolysis constant (day^−1^); the data of lag phase was eliminated before fitting.

The modified Gompertz model, as shown in Equation (7), was proposed to fit the cumulative methane production results obtained from the AD experiments to predict the methane potential (Lay et al., 1998).

(7) $$\begin{array}{l} M\left(t\right)=M_{\text{max}}\cdot \text{exp}\left\{-\text{exp}\left[\frac{R_{\max}\;\cdot \;e}{M_{\max}}\cdot \left(\lambda -t\right)+1\right]\right\} \end{array}$$

where *M*(*t*) denotes the cumulative methane production [ml·(g-VS)^−1^] at AD time *t* (days), *M*_max_ denotes the maximum cumulative methane production potential [ml·(g-VS)^−1^], *R*_max_ denotes the maximum methane production rate [ml·(g-VS)^−1^·day^−1^], *e* is Euler's constant (2.7183), and λ is the lag phase (days).

### Energy Balance

Energy balance is necessary before and after adding the biological pretreatment steps.

(8) $$\begin{array}{l} \Delta \text{E}={\text{E}}_{\text{out}}-{\text{E}}_{\text{in}}-{\text{E}}_{\text{escape}} \end{array}$$

(9) $$\begin{array}{l} {\text{E}}_{\text{out}}=E_{out}^{pretreament}+E_{out}^{digestion} \end{array}$$

(10) $$\begin{array}{l} {\text{E}}_{\text{in}}=E_{in}^{pretreament}+E_{in}^{digestion} \end{array}$$

(11) $$\begin{array}{l} E_{in}^{pretreament}=\frac{c\times \left(T\;final-T\;initial\right)\times V\times \rho }{3600} \end{array}$$

(12) $$\begin{array}{l} {\text{E}}_{\text{escape}}=E_{escape}^{pretreatment}+E_{escape}^{digestion} \end{array}$$

(13) $$\begin{array}{l} E_{escape}^{pretreatment}=\frac{c\times \left(T\;pretreatment-T\;anbient\right)\times V\times \rho }{3600} \end{array}$$

(14) $$\begin{array}{l} E_{escape}^{digestion}=\frac{c\times \left(T\;digestion-T\;anbient\right)\times V\times \rho }{3600} \end{array}$$

where Δ*E* = net energy, kWh; *E*_out_ = output energy, kWh; *E*_in_ = input energy, kWh; *c* = specific heat capacity of water, 4.18 kJ·kg^−1^·°C ^−1^; *T*_final_ = pretreatment temperature, 55°C; *T*_initial_ = the initial temperature of the material, 25°C; *V* = volume of pretreatment; ρ = density of pretreatment liquid, 1,050 kg·(m^3^)^−1^

(15) $$\begin{array}{l} E_{in}^{digestion}=E_{in}^{filling}+E_{in}^{mixing}+E_{in}^{recycling}+E_{in}^{CHP} \end{array}$$

(16) $$\begin{array}{l} E_{out}^{pretreament}=\frac{c\times \left(T\;pretreatment-T\;digestion\right)\times V\times \rho }{3600} \end{array}$$

where *c* = specific heat capacity of water, 4.18 kJ·kg^−1^·°C ^−1^; *T*_pretreatment_ = pretreatment temperature, 55°C; *T*_digestion_ = digestion temperature, 35°C; *V* = the volume of digestive liquid; ρ = the density of the digestive liquid, 1,050 kg·(m^3^)^−1^; $E_{in}^{filling}$ is the energy required to fill the material into the reactor, 3.8 W·(m^3^)^−1^; $E_{in}^{mixing}$ is the electricity required for the mixture in the AD process, 3.8 W·(m^3^)^−1^; $E_{in}^{recycling}$ is the electricity required by the heat pump for water circulation, 2.4 W·(m^3^)^−1^; and $E_{in}^{CHP}$ is the energy consumed by combined heat and power unit (CHP), 74 W·(m^3^)^−1^ (Dahunsi et al., 2017; Sagarika et al., 2020).

(17) $$\begin{array}{l} E_{out}^{digestion}\;=E_{out}^{heat}\;+E_{out}^{electricity} \end{array}$$

(18) $$\begin{array}{l} E_{out}^{heat}=E_{boiler}^{heat}+E_{CHP}^{heat} \end{array}$$

(19) $$\begin{array}{l} E_{boiler}^{heat}=0.05Y_{\text{yield}}m_{\text{vs}}\zeta \eta_{boiler}^{heat} \end{array}$$

(20) $$\begin{array}{l} E_{CHP}^{heat}=0.9Y_{\text{yield}}m_{\text{vs}}\zeta \eta_{CHP}^{heat} \end{array}$$

(21) $$\begin{array}{l} E_{out}^{electricity}=0.9Y_{\text{yield}}m_{\text{vs}}\zeta \eta_{CHP}^{electricity} \end{array}$$

where *Y*_yield_ = methane production, ml·g-VS^−1^ or m^3^·ton-VS^−1^; *m*_VS_ = feedstock mass, a metric ton of volatile solids, ton-VS^−1^; ζ = lower heating value of methane, 35.9 MJ·(m^3^)^−1^; $\eta_{boiler}^{heat}$, $\zeta \eta_{CHP}^{heat},$ and $\eta_{CHP}^{electricity}$ represent the energy conversion efficiency for the boiler heat and heat and electricity of CHP, 85, 55, and 30%, respectively (Pavlo et al., 2018).

### Statistical Analysis

All analyses were performed in triplicate. Data were presented as mean values and standard deviations and processed using Excel 2016. The kinetic models were fitted using Origin 9.1.

### Analytical Methods

Soluble chemical oxygen demand (sCOD), TS, VS, alkalinity (ALK), total volatile fatty acids (VFAs), and total ammonia nitrogen (by phenate method) were evaluated according to a standard method (American Public Health Association, 2005). The elemental composition (C, H, N, and O) of each substrate was assessed using an elemental analyzer (Vario EL cube, elementar, Germany); results were reported as a percentage of dry weight. Acetic, propionic, and butyric acids were determined by HPLC (ICSep COREGEL 87H3 Column, the HPLC detector was UV 210) under the following conditions: Flow rate = 0.6 ml min^−1^; temperature = 60°C, and the mobile phase = 0.008 N H_2_SO_4_. Total nitrogen was measured according to the method described by Kjeldahl (Metcalf et al., 2002).

## Results and Discussion

### Biological Pretreatment Results

#### Substrate Biological Pretreatment Results

The substrate properties of AD are shown in Table 3. After biological pretreatment, the content of cellulose, hemicellulose, and lignin was reduced by 62.20, 59.58, and 33.77%, respectively. The concentrations of total sugar, sCOD, and VFAs in the pretreatment solution were greatly improved, showing 349.71, 142.73, and 34.83% increase, respectively. This can be attributed to the decomposition of cellulose, hemicellulose, and lignin in the substrate that are difficult to hydrolyze naturally by the cellulolytic microflora in the pretreatment stage. In addition, the concentration of butyric acid in the pretreatment solution was 19.72 times more than that without bio-pretreatment. This indicates that the biological pretreatment of RS and PM may be a metabolic process dominated by butyric acid production. Accumulated butyric acid is rapidly utilized by acetogenic bacteria in the AD stage. Consistent with the work of Caixia and Yebo (2010), metabolites such as organic acids and sugars accumulated in the pretreatment stage are rapidly used by methanogens to shorten the lag period of the AD process.

**Table 3:** Anaerobic digestion substrate properties.

	**Experimental group**	**Control group**
Weight loss rate (%)	39.4 ± 1.06	0
sCOD (mg/L)	7860.3 ± 244.12	3238.24 ± 123.6
Total sugar (mg/L)	908.33 ± 38.68	201.98 ± 9.19
VFAs (mg/L)	600 ± 48	445 ± 34
Acetic acid (mg/L)	213.39 ± 10	150.89 ± 12
Propionic acid (mg/L)	33.53 ± 3.2	10.31 ± 0.5
Butyric acid (mg/L)	255.38 ± 10	12.95 ± 0.5
Cellulose (%)	10.95 ± 0.16	28.97 ± 0.38
Hemicellulose (%)	11.03 ± 0.08	27.29 ± 0.27
Lignin (%)	4.02 ± 0.02	6.07 ± 0.04

#### Methane Production Potential and Biodegradability

**Figure 1:**
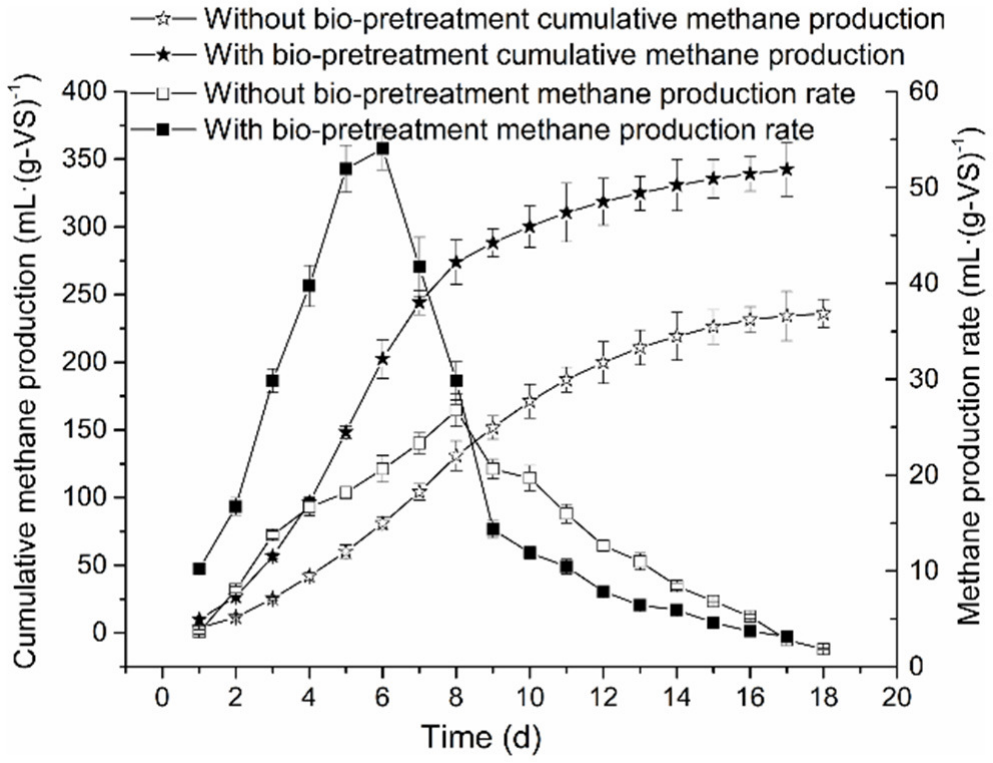
Comparison of cumulative methane production and methane production rate of RS and PM anaerobic co-digestion.

A comparison of the cumulative methane production and methane production rate of anaerobic co-digestion of RS and PM mixtures with or without biological pretreatment under the maximum OLR conditions is illustrated in Figure 1.

Both the cumulative methane production curves in the experimental and control groups were accorded with the sigmoidal shape; this is similar to the growth curve of methanogens. Therefore, these curves were considered suitable in the application of the modified Gompertz model. Similarly, the process kinetic parameters of AD are mainly affected by hydrolysis and methanogenesis (Taricska et al., 2011). Besides, the cumulative methane production of RS and PM after biological pretreatment (experimental group) reached 342.35 ml·(g-VS)^−1^, which was 45% higher compared to that of the control group [236.03 ml·(g-VS)^−1^]. This may be attributed to highly accumulated nutrients in the system after biological pretreatment. These nutrients are rapidly utilized by methanogens so that the methane production in the AD system after biological pretreatment and the bacterial activity are higher than that without biological pretreatment. Also, the lignocellulose structure in the straw and PM in the experimental group system is greatly destroyed; thus, it becomes difficult to prevent microorganisms from attacking the inside. The above findings further demonstrated that the biological pretreatment exerts a productive effect on methane production of RS and PM mixtures.

Moreover, we found that in both the experimental and control groups, after a specific duration, the methane production rate is relatively lower in the early stage of the AD process, which might represent the lag phase. Compared with the experimental group (6 days), this duration of time of the control group (8 days) lasted longer. In the present study, the methane production rate of RS and PM mixtures was at maximum [54.02 ml·(g-VS)^−1^ day^−1^] on the 6th day after being pretreated with the cellulolytic microflora. In comparison, the maximum methane production rate of the control group was 26.76 ml·(g-VS)^−1^·day^−1^, on the 8th day. The above findings imply that biological pretreatment destroys the glycosidic bonds in lignocellulose and improves the hydrolysis efficiency of the substrate; thus, microorganisms are more likely to attack the inside of the substrate. In addition, sugars and VFAs are highly concentrated in the pretreatment stage. Therefore, after the pretreatment of RS and PM, the acid-producing microorganisms in the system can quickly adapt to the environment. Methanogens exhibit a higher rate of methane production after they advance past the lag period. We reported that the performance of the experimental group was better than that of the control group. Besides, methane production was believed as the most intuitive indicator that can reflect the efficiency of AD. Notably, the cellulolytic microflora proposed in the present study can effectively improve the AD effect of RS and PM mixture and shorten the lag phase. This might be attributed to the recalcitrant lignocellulosic structures of RS and PM substrates, which can hardly be absorbed and utilized by methanogens, thus resulting in the lower methane production velocity. However, in the experimental group, pretreatment with the cellulolytic microflora could break down the complex lignocellulosic structures, generating sugar, organic acids, and other nutrients that could be easily utilized by methanogens. Therefore, the methanogenesis was accelerated and further enhanced methane production. Previous reports believed that the biological pretreatment process can further benefit the AD and improve the final anaerobic efficiency (Park et al., 2009).

Besides, it was found that after biological pretreatment, the maximum biodegradability predicted by the modified Gompertz models was 68.35%, whereas the actual maximum biodegradability was 66.70%. In AD without bio-pretreatment, the above two values were 47.12 and 45.99%, respectively. This indicates that the AD performance of RS and PM is improved after compound microbial pretreatment, an observation that is consistent with the work of Uma et al. (2013).

Wang S. Q. et al. (2018) used cellulase produced by *Aspergillus niger* to pretreat corn Stover at 50°C for 60 h, and the subsequent AD increased methane production by 36.9% compared with the unpretreated substrate. Fu et al. (2016) used microaerobic bacteria to pretreat effluent from retted corn straw at 55°C and 130 rpm, which improved the methane yield by 21% and the VS removal rate by 10% during AD. This indicates that the pretreatment of lignocellulosic materials using cellulose-degrading microflora has a great application prospect.

#### Comparison and Analysis of Kinetic Parameters

**Figure 2:**
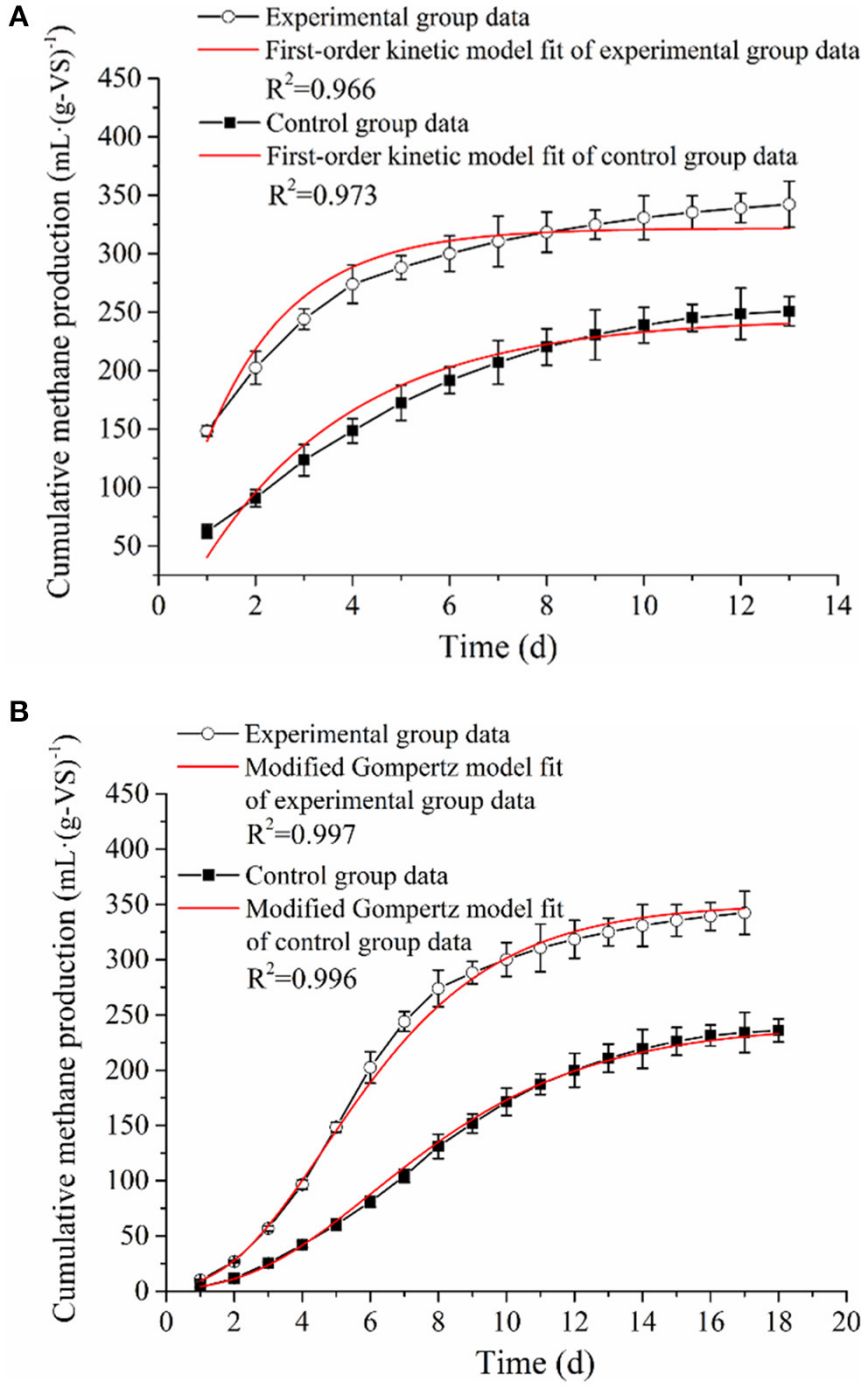
The **(A)** first-order kinetic model and **(B)** modified Gompertz model fitting curves of RS and PM anaerobic co-digestion.

The kinetic parameters of the AD process help to understand the system evolution of the fermentation process. In the present study, the first-order and modified Gompertz models were proposed to fit the accumulative methane production data observed from the AD experiments with or without bio-pretreatment. The estimated kinetic parameters and model fitting curves are shown in Table 4 and Figure 2. The correlation coefficients (*R*^2^) of the experimental (0.997) and control (0.996) groups revealed that the modified Gompertz models have high correlations and are more suited in simulating the methane production and estimating the lag phase (Table 2). Generally, the maximum cumulative methane production potential (*M*_max_) and the maximum methane production rate (*R*_max_) can directly reflect the efficiency of an AD. In the present study, the *M*_max_ and *R*_max_ of RS and PM after biological pretreatment reached 350.79 ml·(g-VS)^−1^ and 45.36 ml·(g-VS)^−1^·day^−1^, which were 45.1 and 84.3% higher compared to those of the control group, respectively.

**Table 4:** Kinetic parameters of anaerobic digestion with or without bio-pretreatment.

	**Modified Gompertz model**				**First-order model**			
	***R*_**emix**_ ml·(g-VS)^**−1**^**·**day^**−1**^**	***M*_**max**_ ml·(g-VS)^**−1**^**	**λ (days)**	***T*_**90**_ (days)**	***R*^**2**^**	***M*_**max**_ ml·(g-VS)^**−1**^**	***k* (day^**−1**^)**	***R*^**2**^**
Experimental group	45.36 ± 0.74	350.79 ± 6.2	1.79 ± 0.03	8.92 ± 0.02	0.997	321.78 ± 6.81	0.46 ± 0.03	0.966
Control group	24.60 ± 0.48	241.82 ± 6.1	2.43 ± 0.05	10.7 ± 0.04	0.996	231.13 ± 8.95	0.26 ± 0.02	0.973

*All data are shown as means ± standard deviations (n = 3)*.

Moreover, it was observed that the RS and PM anaerobic co-digestion in the experimental and control groups have an obvious lag phase (λ) of 1.79 and 2.43 days, respectively. Generally, the λ value indicated the time required for methanogens to adapt to the substrates before producing methane (Syaichurrozi et al., 2016). The lower λ value implied that a shorter duration is required to generate methane. As shown in Table 3, the λ value in the experimental group with the pretreated RS and PM mixture as the feedstuff was 1.79 days, which was shorter than that of the control group (2.43 days). This concurred with the findings on the peak gas production time of the experimental group, which occurred earlier than that of the control group in Figure 1. A study by Dahunsi et al. also obtained similar results in the pretreatment of lignocellulosic biomass (Dahunsi, 2019). The above results can be attributed to the cellulolytic microflora attacking the rigid structure in cellulose during the pretreatment stage, making it easier for acid and gas-producing microorganisms to use the substrate, thereby improving the utilization efficiency of the substrate. Consequently, the proliferation rate of methanogens is elevated, and the eventual reduction of the lag phase occurs.

Meanwhile, another important kinetic parameter, that is, effective methane production time (*T*_90_), was, in most cases, used to predict the duration of AD and methane production. The value of *T*_90_ was calculated by subtracting λ from the time required to attain 90% of the methane production. Notably, the *T*_90_ of RS and PM mixtures with or without bio-pretreatment was 8.92 and 10.7 days in the experimental and control groups, respectively (Table 4). The above observation revealed that the AD period of the experimental group was shorter compared to that of the control group. Further, through combined analysis of the maximum cumulative methane production potential [350.79 ml·(g-VS)^−1^] and maximum methane production rate [45.36 ml·(g-VS)^−1^·day^−1^], a shorter lag phase, *T*_90_, and higher methane production rate were obtained in the experimental group, indicating that pretreating RS and PM with the cellulolytic microflora could accelerate the AD process and generate more methane at a faster rate.

Furthermore, the hydrolysis constant (*k*) parameter could be applied to evaluate the process rate-limiting stage and estimate the substrate suitability. Meanwhile, *k* value describes the degradation rate and the production of methane; in other words, higher *k* value signifies higher degradation and methane production (Li et al., 2016). In this study, the AD data from the experimental and control groups were efficiently fitted using the first-order model (both their *R*^2^-values were over 0.966). We also observed that the *k* value of the RS and PM mixture after bio-pretreatment was 0.46 day^−1^, whereas it was 0.26 day^−1^ in the control group. The above results revealed an increase in the hydrolysis rate of RS and PM by 77% after pretreatment with a cellulolytic microflora. It was believed that the cellulosic components of RS and PM mixtures are difficult to be degraded for their smooth surfaces and compact structures. After biological pretreatment, these dense structures are destroyed, and their contact with hydrolytic bacteria is enhanced. Thus, the structures can easily be broken down by hydrolytic enzymes, which increases the hydrolysis efficiency and *k*-value (Zhang et al., 2016; Rodriguez et al., 2017).

#### Analysis of the Process Parameters and Their Correlations With Methane Production and Kinetic Parameters: pH, Alkalinity, and Volatile Fatty Acids

pH is considered as a key indicator that can reflect the proceeding condition of an AD system. Notably, a pH range of 6.8–7.4 has been reported as most suitable for the growth of methanogens (Li et al., 2016). Additionally, in an AD process, alkalinity (ALK) can neutralize the excessive accumulation of VFAs to stabilize the pH and thus alleviate its inhibitory effect on methanogens (Ripley et al., 1986; Hawkes et al., 1994). Therefore, these parameters are critical process parameters always observed in an anaerobic system.

**Figure 3:**
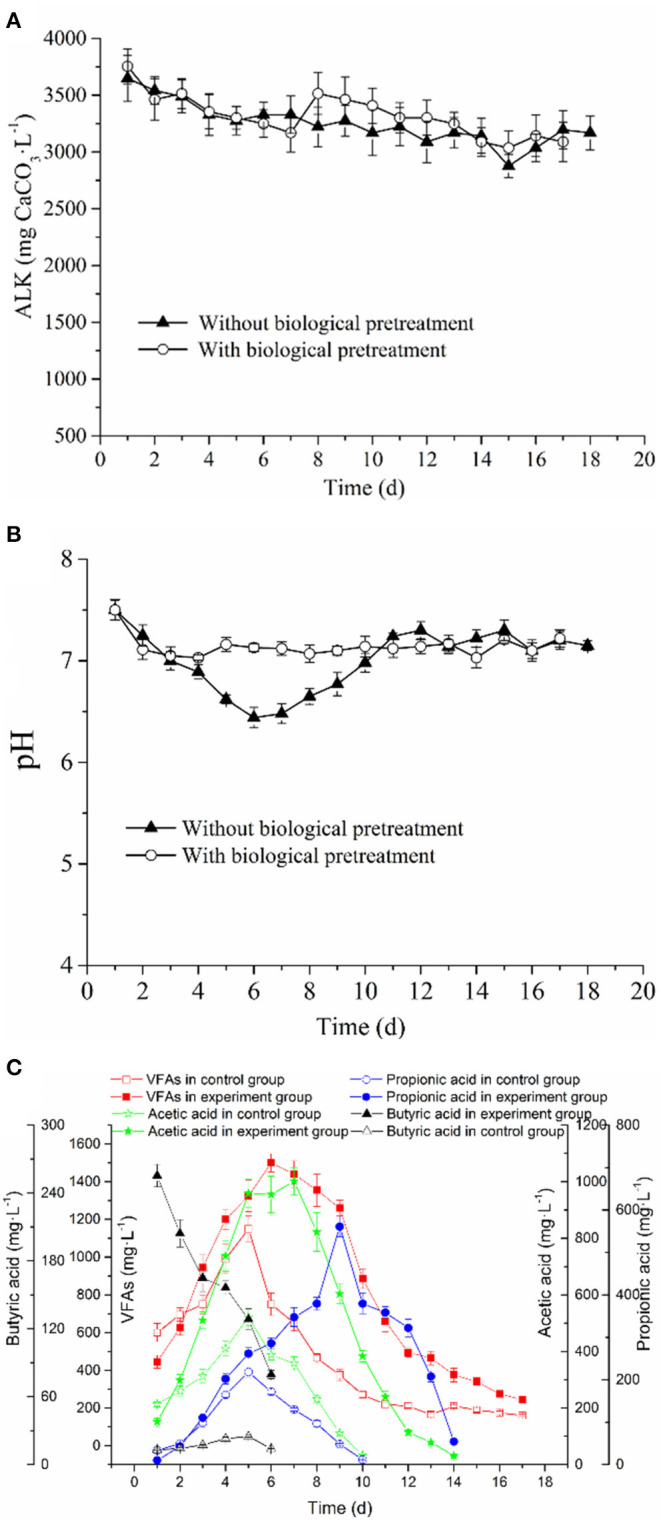
Changes of **(A)** ALK, **(B)** pH and VFAs of RS and PM without or with **(C)** biological pretreatment in the anaerobic co-digestion (*p* < 0.05).

Herein, the ALK in the AD system of the control and experimental groups slowly decreased from about 3,647–3,168 and 3,754–3,088 mg CaCO_3_·L^−1^, respectively (Figure 3A). These ALK values indicated that both the experimental and control AD systems have high buffering capacities. Besides, the pH value of the experimental and control groups showed the same declining tendency in the early stage of the AD process (Figure 3B), which may be attributed to the accumulation of VFAs. However, in the late stage, as the methanogens continuously consume VFAs, the pH value gradually increases and is later stabilized. In this work, the pH range of the experimental group in the whole AD process generally was maintained above 7.05. However, the pH in the control group rapidly dropped to about 6.45 in the early anaerobic stage, which was lower than the optimal pH range required by methanogens for growth (6.8–7.4). Because methanogens are sensitive to changes in pH, the pH of the control group in this period potentially impacted the growth of methanogens, which further result in the transition to the lag phase. The above analysis showed that the duration of the lag phase might be related to pH value, in that, a lower pH value would prolong the adaptation time for methanogens to environmental changes, which eventually results in a longer lag phase. Additionally, it was found that the period of rapid decrease of pH in the control group corresponded to that of the increase of methane production rate (see Figures 1, 3). However, the methane production rate was low. This indicated that lower pH potentially affected the maximum methane production rate, thereby reducing the cumulative methane production.

The change in pH is always believed to be associated with the variation of VFAs; therefore, its influence on the lag phase of AD might be caused by the accumulation of VFAs. It is particularly necessary to evaluate the changes in the concentration and composition of VFAs during an AD process. In the hydrolysis phase of the AD process, the lytic monomers or dimers were further transformed into VFAs such as formic, acetic, propionic, and butyric acid, which could be used by methanogens to generate methane. However, the excessive accumulation of these intermediate metabolites would limit methanogenesis due to thermodynamic inhibitions (Xiao et al., 2013). The VFAs of the experimental and control groups were accumulated in the initial stages and were mainly composed of acetic and propionic acids (Figure 3C). Notably, acetic acid was regarded as the precursor that can be directly utilized by methanogens, whereas highly concentrated propionic acid exerted a strong toxic effect on methanogens. It was always believed that when the concentration of propionic acid reaches over 1,000 mg·L^−1^, the AD process is inhibited (Hanaki et al., 1994). However, the low propionic acid concentration is believed to possess some benefits for methane fermentation (Yuan et al., 2012). Based on Figure 3C, the maximum propionic acid concentration in the control and experimental groups both do not exceed the tolerance concentration. Thus, with the consumption of acetic and propionic acids in the experimental and control groups, the VFA concentration decreased. Besides, the concentration curve of butyric acid was different from other organic acids (Figure 3C). Since PM contains a small amount of organic acid, mainly butyric acid, traces of butyric acid can be detected in the initial stage of the control group (Ni et al., 2012). A large amount of butyric acid in the initial phase of the experimental group was produced via the degradation of RS and PM by the cellulolytic microflora during the pretreatment stage. In the AD stage, the butyric acid was rapidly consumed and disappeared on the 6th day; similar results were reported by Fernan et al. (2017).

In the methanogenesis phase, it was believed the accumulation of VFAs would greatly inhibit the growth of methanogens (Xiao et al., 2013). Notably, we found that the accumulation of VFAs in the control group was higher than that in the experimental group (Figures 1, 3C); this inhibited the activity of some methanogens in the AD system. As a result, methanogens were characterized by a decrease in the utilization efficiency of acetic acid, which explains why the cumulative methane production and methane production rate in the control group were lower than those in the experimental group. Furthermore, in the AD process of the experimental and control groups, lag phases could be observed, and propionic acids were accumulated in this period, indicating that the occurrence of the lag phase may be associated with the accumulation of propionic acids. This assumption could also be proved by the following phenomena observed from Figure 3C: (1) In the middle stage of the AD process, the degree of accumulation of VFAs in the control group was significantly higher than that in the experimental group, which may be the primary cause of the prolonged period; this result is consistent with a report from the literature (Mao et al., 2017). (2) The time when VFA concentration of the experimental and control groups began to decrease coincided with the time at which methane production was at the peak; however, with the decrease in VFAs, the growth rate of the cumulative methane production rate of the experimental group and the control group was lowered. (3) The peaks of the control group and the experimental group occurred when acetic acid accumulated to the maximum, indicating that *R*_max_ and *T*_90_ may be related to the maximum concentration of acetic acid. In subsequent research or application, some methods can be adopted to increase the concentration of acetic acid in the AD system, thus shortening the *T*_90_, which is highly vital for the maximum utilization of equipment. Findings by Li H. L. et al. (2016) showed that at an acetic acid concentration of <120,000 mg/L, the rate at which methanogens utilize acetic acid is positively correlated with the acetic acid concentration, and this correlation will be directly reflected in the methane production rate, which is consistent with the above results.

#### Analysis of Process Parameters and Their Correlations With Methane Production and Kinetic Parameters: Ammonia Nitrogen

**Figure 4:**
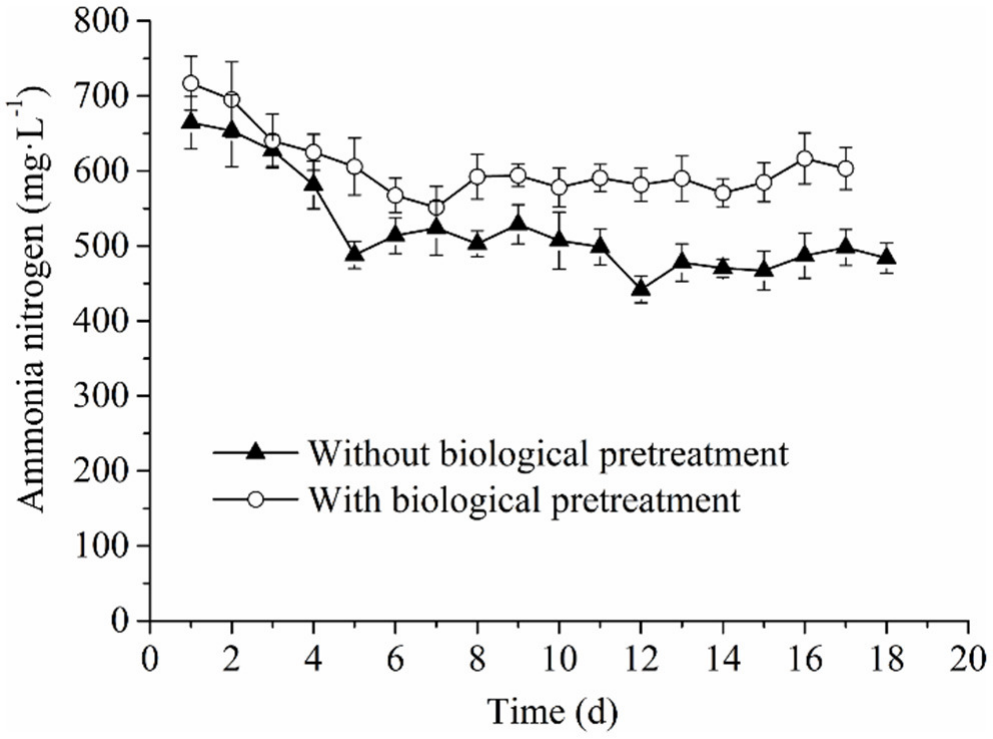
Changes of ammonia nitrogen in the anaerobic co-digestion process.

The ammonia nitrogen concentration in the experimental and control groups both revealed decreased tendencies with the prolonged AD process, which then stabilized in the late stage (Figure 4). Furthermore, it was found that the concentration of ammonia nitrogen in the experimental group was always higher than that of the control group. This could be explained by the fact that in the pretreatment process, more proteins and amino acids were converted into ammonia nitrogen by the cellulolytic microflora during hydrolysis. On the other hand, in the AD process, RS and PM mixtures would still be slowly hydrolyzed, and the existence of more hydrolytic microorganisms from the cellulolytic microflora maintained the production rate of ammonia nitrogen in the experimental group than that of the control group. Subsequently, a higher concentration of ammonia nitrogen in the fermentation solution of the experimental group was reported. According to previous reports, the ammonia nitrogen concentration at 50–200 mg·L^−1^ can promote the growth of microorganisms, whereas ammonia nitrogen concentration at 200–1,000 mg·L^−1^ exerts no antagonistic effect. However, when the concentration reaches 1,500–10,000 mg·L^−1^, the activity of microorganisms is inhibited (Rajagopal et al., 2013; Sung and Liu, 2013). In this study, the ammonia nitrogen concentration in the experimental and control groups ranged between 200 and 1,000 mg·L^−1^, which indicated that although the ammonia nitrogen production of the experimental group was higher than that of the control, it could not reduce the benefit of higher methane production. Therefore, the ammonia nitrogen concentration in the AD system could not prolong or shorten the AD lag phase by impacting the activity of methanogens. Meanwhile, it exerted no significant effect on other kinetic parameters (*k, T*_90_, *R*_max_, and *M*_max_). Herein, we suggested that the greater significance of ammonia nitrogen concentration may be the main alkaline substance to maintain ALK, thus ensuring that the anaerobic system would not be acidified (Speece, 1983).

#### Analysis of Process Parameters and Their Correlations With Methane Production and Kinetic Parameters: sCOD Removal

**Figure 5:**
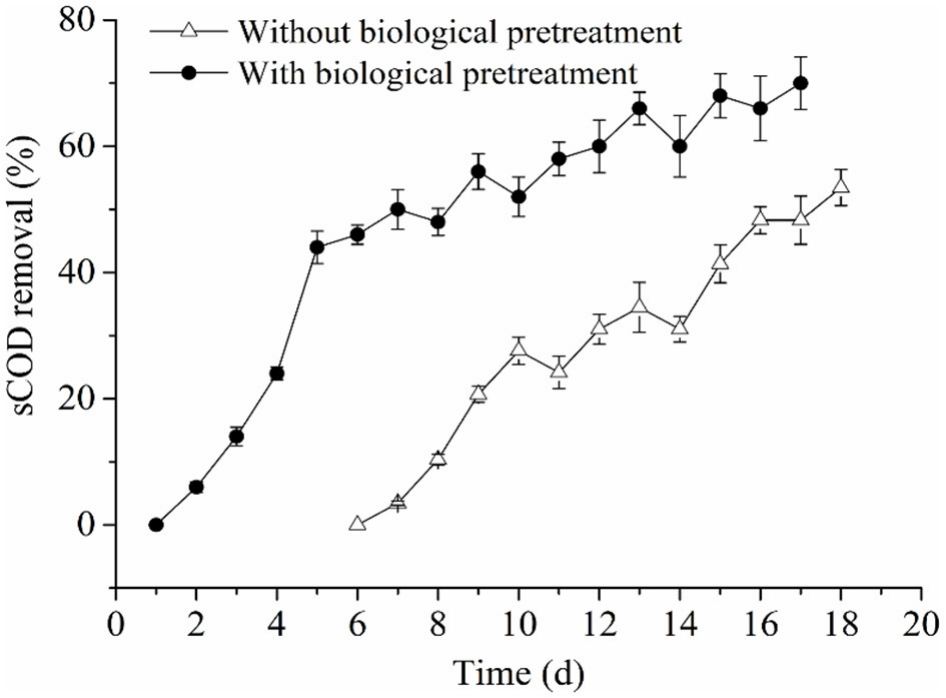
Changes of sCOD removal in the RS and PM anaerobic co-digestion.

In an AD system, methanogens can transform some soluble organic substrates into methane. Therefore, the parameter of sCOD (soluble chemical oxygen demand) removal can indicate methanogenic activity. In this work, sCOD removal in the experimental and control groups both showed increased trends (Figure 5). However, sCOD removal in the experimental group reached 70%, while that of the control was 53.4% when the AD process was ceased. Besides, it is worth noting that we did not calculate sCOD removal in the control group during the first 6 days of this study due to the slow consumption of sCOD during this period. The reason for this phenomenon might be explained by non-pretreatment of the RS and PM in the control group, such that lignocellulosic components were slowly decomposed into sugars, alcohols, and organic acids, by hydrolyzing microbes. These substances could directly be utilized by methanogens when they are further converted into acetic acid through the activity of acetic acid-producing bacteria. However, due to the tight and complex structure of lignocellulose, this conversion rate is very slow. Therefore, in the early stage, the activity of methanogens was lower than that of hydrolyzing bacteria because of the low levels of available nutrients that could be utilized by methanogens. Thus, the generation of soluble organic matter exceeded the consumption of methanogens, and sCOD was accumulated. After 6 days of incubation, as the methanogens adapted to the environmental conditions and the acetic acid content increased, the activity of methanogens was gradually higher than the hydrolyzing bacteria. Also, a large amount of organic matter was utilized by methanogens to produce methane, which further led to an increase in the sCOD removal ratio.

In the experimental group, a large amount of organic matter, mainly composed of VFAs, were produced from RS and PM with biological pretreatment, which could be rapidly consumed by methanogens in the early stage of the AD process. Therefore, more methane was produced in the early stage, and the AD lag phase and duration in the experimental group were shortened. In addition, we found that the sCOD removal in the experimental group was significantly higher compared to that of the control group; these findings concurred with their accumulative methane production and methane production rate. Further, the above observations revealed that sCOD removal is positively correlated with *R*_max_ and *M*_max_, which was consistent with reports from previous studies (Syaichurrozi and Budiyono, 2013; Li H. L. et al., 2016).

#### Energy Balance

Bio-pretreatment is an energy-consuming process, whereas AD is an energy production process; therefore, it is important to maintain the energy balance of the bio-pretreatment followed by AD technique. Generally, the additional energy required for bio-pretreatment should be included in the AD process after bio-pretreatment. In this work, to fully evaluate the energy balance and economic feasibility of biological pretreatment, the combined heat and power (CHP) system was adopted with thermal and electrical efficiencies at 50 and 35%, respectively (Table 5), which is a highly popular technique in energy conversion across the globe (Lay et al., 1998; Dahunsi et al., 2017). We used the bio-pretreatment method to pretreat RS and PM, which was followed by anaerobic co-digestion. For each ton of material processed, compared to without bio-pretreatment, only 492.73 kWh extra energy was consumed, which could increase the energy output by 5133.02 kWh after AD. Similarly, in previous studies where agricultural waste was subjected to bio-pretreatment methods, positive energy gains were obtained (Yin et al., 2016). Moreover, pretreatments using materialization methods generated similar results (Liang et al., 2016; Kovačić et al., 2019), which showed that in terms of energy gains, pretreatment of materials before AD has great application prospects.

**Table 5:** Energy evaluation of anaerobic co-digestion of RS and PM.

**Project**	**$E_{\text{in}}^{\text{pretreatment}}$**	**$E_{\text{in}}^{\text{filling}}$**	**$E_{\text{in}}^{\text{mixing}}$**	**$E_{\text{in}}^{\text{recycling}}$**	**$E_{\text{out}}^{\text{pretreatment}}$**	**$E_{\text{boiler}}^{\text{heat}}$**	**$E_{\text{CHP}}^{\text{heat}}$**	**$E_{\text{out}}^{\text{electricity}}$**	***E*_**escape**_**	**Net energy**
Control group	–	16.57	16.57	10.51	–	657.27	7654.54	4175.15	123.13	12320.18
Experiment group	369.49	16.57	16.57	10.51	246.26	953.33	11103.12	6056.26	492.63	17453.20

*Includes heat energy generated but not used at the time of the study*.

*Unit: kWh*.

*Calculated by 1 ton of raw material (pig manure and rice straw)*.

## Conclusion

In the present study, the methane yield and kinetic parameters of RS and PM anaerobic co-digestion with or without bio-pretreatment showed significant variations. After bio-pretreatment, the cumulative methane production of RS and PM was recorded at 342.35 ml·(g-VS)^−1^, which was a 45% increase, while the biodegradation increased by 45.06%. Based on results generated by the kinetics models, bio-pretreatment improved the hydrolysis constant, maximum cumulative methane production potential, and the maximum methane production rate, but shortened the lag phase and effective methane production time. Regarding energy balance, biological pretreatment only consumes 738.99 kWh·ton^−1^, but 5133.02 kWh·ton^−1^ can be obtained in the AD stage, compared to when non-bio-pretreated materials are used. This work demonstrated that bio-pretreatment with a cellulolytic microflora could effectively improve the methane yield of RS and PM anaerobic co-digestion and is an environmentally friendly mechanism. It also could provide technical reference for kinetics and anaerobic co-digestion parameters of RS and PM.

## Data Availability Statement

The original contributions presented in the study are included in the article/supplementary material, further inquiries can be directed to the corresponding author/s.

## Author Contributions

BZ, FS, XA, and QZ: conceptualization, writing—review, and editing. BZ, FS, and WA: methodology and formal analysis. BZ and FS: writing—original draft. QZ: funding acquisition and supervision. All authors contributed to the article and approved the submitted version.

## Conflict of Interest

The authors declare that the research was conducted in the absence of any commercial or financial relationships that could be construed as a potential conflict of interest.

## Abbreviations

RS: rice straw
PM: pig manure
C/N: carbon/nitrogen ratio
AD: anaerobic digestion
ADM1 model: Anaerobic Digestion Model No.1
TS: total solids
VS: volatile solids
MS: mixed substrates
PCS: peptone–cellulose solution
CBPs: cellulose-binding proteins
OLR: organic loading rate
COD: chemical oxygen demand
CK: blank groups
TBMP: theoretical biochemical methane potential
λ: lag phase
*k*: hydrolysis constant
sCOD: soluble chemical oxygen demand
ALK: alkalinity
VFAs: volatile fatty acids
HPLC: high-performance liquid chromatography.
